# Gene activation-associated long noncoding RNAs function in mouse preimplantation development

**DOI:** 10.1242/dev.116996

**Published:** 2015-03-01

**Authors:** Nobuhiko Hamazaki, Masahiro Uesaka, Kinichi Nakashima, Kiyokazu Agata, Takuya Imamura

**Affiliations:** 1 Department of Biophysics and Global COE Program, Graduate School of Science, Kyoto University, Kitashirakawa-Oiwake, Sakyo-ku, Kyoto 606-8502, Japan; 2 Division of Basic Stem Cell Biology, Department of Stem Cell Biology and Medicine, Graduate School of Medical Sciences, Kyushu University, 3-1-1 Maidashi, Higashi-ku, Fukuoka 812-8582, Japan

**Keywords:** Long noncoding RNA, DNA demethylation, Early mouse preimplantation development, Zygotic gene activation, Epigenetic regulation, Pluripotency

## Abstract

In mice, zygotic activation occurs for a wide variety of genes, mainly at the 2-cell stage. Long noncoding RNAs (lncRNAs) are increasingly being recognized as modulators of gene expression. In this study, directional RNA-seq of MII oocytes and 2-cell embryos identified more than 1000 divergently transcribed lncRNA/mRNA gene pairs. Expression of these bidirectional promoter-associated noncoding RNAs (pancRNAs) was strongly associated with the upregulation of their cognate genes. Conversely, knockdown of three abundant pancRNAs led to reduced mRNA expression, accompanied by sustained DNA methylation even in the presence of enzymes responsible for DNA demethylation. In particular, microinjection of siRNA against the abundant pancRNA partner of interleukin 17d (*Il17d*) mRNA at the 1-cell stage caused embryonic lethality, which was rescued by supplying IL17D protein *in vitro* at the 4-cell stage. Thus, this novel class of lncRNAs can modulate the transcription machinery in *cis* to activate zygotic genes and is important for preimplantation development.

## Abstract (precis)

**Highlighted article:** During zygotic gene activation in the early mouse embryo, promoter-associated noncoding RNAs can promote expression of their partner mRNAs, which may be important for normal development.

## INTRODUCTION

Transcription of the zygote genome begins mainly at the 2-cell stage. Genome-wide gene activation in the zygote, termed zygotic gene activation (ZGA), is regarded as crucial for embryos to acquire the potency to form all cell types. In the mouse, ZGA starts around the pronuclear stage, followed by a major wave of transcription at the 2-cell stage (Aoki et al., 1997; Latham et al., 1991). During ZGA, the patterns of various types of DNA and histone modification are dynamically reconstructed. For example, the sperm genome is tightly packaged by protamines that suppress nascent transcription (Braun, 2001). These sperm-derived protamines are replaced with maternally hyperacetylated histones that allow the onset of nascent transcription in the zygote (Santos et al., 2005). DNA demethylation is another well-known epigenetic event involved in the reconstruction of zygote chromatin structure. It has long been believed that after fertilization the bulk of the DNA, including repeat sequences such as long interspersed nuclear elements (LINEs), becomes demethylated as a major part of epigenetic reprogramming (Farthing et al., 2008; Mayer et al., 2000; Oswald et al., 2000). In actuality, DNA methylation at individual promoter regions differs during the epigenetic reprogramming (Borgel et al., 2010; Smallwood et al., 2011; Smith et al., 2012). The DNA methylation pattern of promoter regions seems to be determined in a gene-specific manner by an equilibrium between DNA methylation and demethylation. Thus, distinct and sequence-specific machineries should regulate this limited program of gene activation. One key issue is how such sequence-specific gene activation is achieved towards the acquisition of pluripotency in early mouse embryos.

Long noncoding RNAs (lncRNAs) constitute one group of factors that can explain such local epigenetic alterations. The number of known lncRNAs is now rapidly increasing, and experimental evidence for epigenetic alterations mediated by long intergenic noncoding RNAs, a fraction of lncRNAs, is accumulating. For example, *HOTAIR* acts as a chromatin repressor at hundreds of promoters with polycomb repressive complex 2 (Gupta et al., 2010; Rinn et al., 2007; Tsai et al., 2010). Another set of lncRNAs transcribed from bidirectional promoters, named promoter-associated noncoding RNAs (pancRNAs), are poly(A)^+^ RNAs involved in the sequence-specific upregulation of their oppositely transcribed partner genes (Imamura et al., 2004b; Tomikawa et al., 2011). Some of these poly(A)^+^ RNAs have been confirmed to induce DNA demethylation in their promoter regions in a sequence-specific manner (Tomikawa et al., 2011). We and another group have also reported that thousands of pancRNAs are generated by transcription of the antisense strand and exhibit expression changes coordinated with their cognate gene. Moreover, pancRNA possesses the potential to enhance partner gene expression in a tissue-specific manner in mouse and chimpanzee brain and heart (Uesaka et al., 2014) and during embryonic stem cell (ESC) differentiation (Sigova et al., 2013).

Now, the directional RNA-seq technique has become powerful enough to be applied to very early stage embryos to see whether RNA-directed gene activation occurs in a significant fraction of genes, not only for cell differentiation but also for the acquisition of pluripotency. Therefore, we have started to analyze such comprehensive data to test the idea that the onset of pancRNA expression at ZGA can also activate partner gene expression in a gene-specific manner. In this study, to identify divergently transcribed pancRNA/gene pairs, we obtained the transcriptome of mouse oocytes and showed that more than 1000 such pairs are expressed at ZGA. By manipulating the abundant transcriptional machineries that involve pancRNA, we showed that pancRNAs are functionally associated with the activation of their partner genes. One such pancRNA for the expression of *Il17d*, a member of the interleukin gene family, was shown to be indispensable for embryonic development.

## RESULTS

### The identification of more than 1000 antisense pancRNAs in mouse preimplantation embryos

**Fig. 1.**
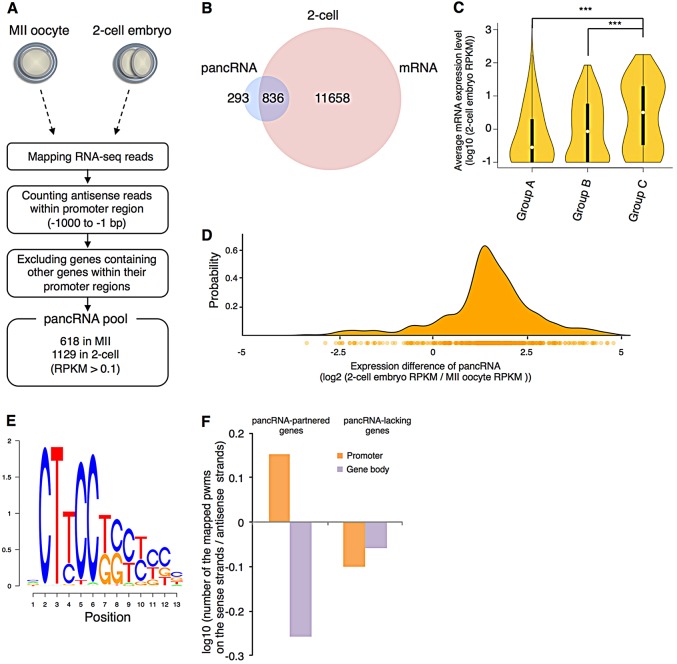
**Characterization of pancRNAs by directional RNA-seq.** (A) Screening of pancRNA datasets. One hundred oocytes or 2-cell embryos were used for each analysis, and four replicates were made for the statistical tests. (B) Numbers of pancRNA and mRNA species present in 2-cell embryos. (C) Violin plot of mRNA levels of 2-cell embryo genes with and without pancRNAs. Groups A, B and C comprise mRNAs without pancRNAs (11658), with the 100 most weakly expressed pancRNAs, and with the 100 most strongly expressed pancRNAs, respectively. Violin width and white circles indicate gene density and median expression levels of mRNAs, respectively. Box plots were merged and are indicated by black bars. ***P<0.001. (D) Expression difference of the pancRNA partners of the upregulated genes and their probability visualized as a density plot. Twofold upregulated genes (which correspond to 520 of the 836 genes in B) were selected. Below the density plot is a unidimensional plot of the expression difference of each pancRNA (circles), in which density is expressed by color intensity. (E) A frequently observed sequence motif in the promoter regions of pancRNA-partnered genes. (F) The frequency of the sequence motif in various gene regions. Sequences with 90% or greater identity to the position weight matrix (pwm) of the motif shown in E were categorized according to their presence on the sense or antisense strand of the promoter (−500 to −1 bp) and gene body (+1 to +500 bp) regions. The TSSs of the pancRNA-partnered genes provide the switching points for the observed asymmetric distribution of the CT-rich sequence.

To examine whether pancRNAs are induced after fertilization, we generated a total of 111 million directional RNA-seq reads using an Illumina HiSeq2000 from mouse metaphase II (MII) oocytes and 420 million directional RNA-seq reads from 2-cell embryos (see Materials and Methods). These reads were mapped to the mouse mm10 genome. Our RNA-seq data showed robust reproducibility among biological replicates (Pearson correlation coefficient, *r*>0.99; supplementary material Fig. S1). 5′-3′ mapping bias was comparable to that of RNA-seq data for oocytes in previous studies (Park et al., 2013) (supplementary material Fig. S2). In order to verify the strandedness of our directional RNA-seq data, we mapped the reads to known RefSeq genes and calculated the proportion that mapped on the correct strand. The results showed that 99.1% of the reads from MII oocytes and 96.9% of the reads from the fertilized 2-cell embryos mapped on the correct strand. Using our high-resolution datasets, we identified 618 and 1129 candidate pancRNAs in MII oocytes and 2-cell embryos, respectively (Fig. 1A).

### Characterization of pancRNA-partnered genes

To test the hypothesis that pancRNAs contribute to the upregulation of their partner genes during ZGA, we examined the expression pattern of the pancRNAs and their mRNAs. We found that 836 of the 1129 pancRNAs were co-expressed with the corresponding mRNAs in 2-cell embryos (Fig. 1B). We further investigated whether the pancRNA/mRNA pairs exhibit coordinated changes of expression, and found that most of the upregulated pancRNA/mRNA pairs showed coordinated upregulation in 2-cell embryos (supplementary material Fig. S3). As shown in Fig. 1C, we compared expression levels among three classes of genes that were expressed in 2-cell embryos: genes whose corresponding pancRNA is not transcribed (Group A), and those whose partner is among the 100 most weakly (Group B) or 100 most strongly (Group C) expressed of the 836 pancRNAs. The average gene expression level correlated with the presence of the respective pancRNA, and the expression levels of the Group C genes were significantly higher than those of Group A and B genes. Moreover, the difference in pancRNA expression level between MII and 2-cell embryos was positively correlated with that of the partner mRNA (Fig. 1D). Interestingly, based on the gene ontology function enrichment analysis, we found that cell death-related genes were enriched among co-upregulated pancRNA-partnered genes (supplementary material Tables S1 and S2). These results support our hypothesis that zygotic pancRNAs are involved in the upregulation of their cognate genes.

To test whether particular sequences are potentially involved in the regulation of pancRNA expression in zygotic pancRNA-partnered genes, we performed *de novo* motif searching and found a CT-rich motif (Fig. 1E; supplementary material Fig. S4A). Since pancRNA-partnered genes frequently contain a CpG island (CpGi) within their promoter region (supplementary material Fig. S4B), we investigated whether CT-rich motifs were enriched within the CpGi-type promoters. We calculated the CT-rich motif frequency within the promoters of CpGi-type and non-CpGi-type genes, and found that the CT-rich motif was present more frequently in the former (56.1% versus 44.8%; supplementary material Fig. S4C). These results suggest that the CT-rich motif is associated with CpGi. Most importantly, the distribution pattern of this CT-rich motif clearly differed between pancRNA-partnered genes and pancRNA-lacking genes (Fig. 1F). In pancRNA-partnered genes, the CT-rich motif was frequently observed on the sense strand of the promoter and on the antisense strand of the gene body. By contrast, in pancRNA-lacking genes, the CT-rich motif was observed on the antisense strand of not only the gene body but also the promoter (supplementary material Fig. S5). This indicates that the transcription start sites (TSSs) of the pancRNA-partnered genes are the switching points for the observed asymmetric distribution of the CT-rich motif.

### Ability of pancRNAs to regulate gene activation

**Fig. 2.**
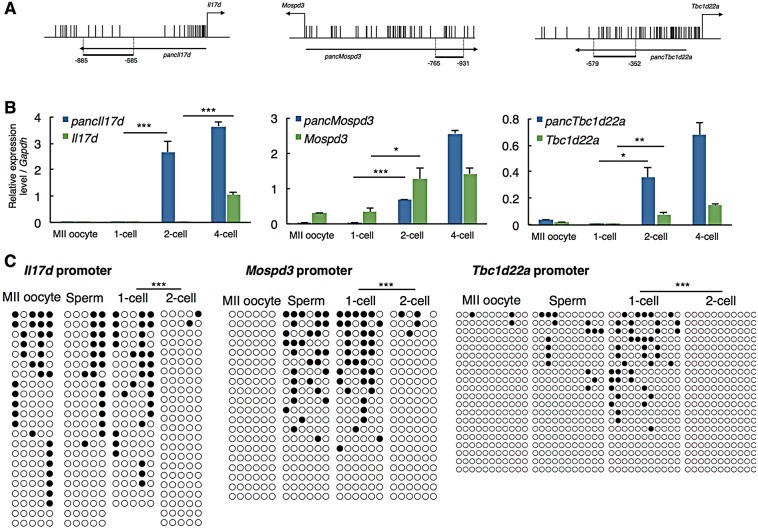
**Effect of pancRNA knockdown on the expression of the counterpart gene during early development.** (A) 5′-regions of mouse *Il17d*, *Mospd3* and *Tbc1d22a*. Vertical lines mark the locations of CpG dinucleotides. Thick horizontal lines denote the regions analyzed by bisulfite sequencing. Primer positions are numbered relative to the TSS of each gene. (B) qPCR analysis of pancRNA and mRNA at the *Il17d*, *Mospd3* and *Tbc1d22a* loci in MII oocytes and in fertilized 1-cell, 2-cell and 4-cell embryos. Error bars indicate s.e.m. (C) DNA methylation levels of the promoter regions of *Il17d*, *Mospd3* and *Tbc1d22a* in MII oocytes, sperm and fertilized 1-cell and 2-cell embryos. Filled and open circles indicate methylated and unmethylated cytosines, respectively. *P<0.05; **P<0.01; ***P<0.001.

To examine the function of the ZGA-associated pancRNA, we selected highly expressed pancRNAs that were upregulated at the 2-cell stage and whose expression was maintained at a high level in ESCs (supplementary material Table S3), and characterized the three most highly expressed in ESCs, namely those partnered with *Il17d* (*pancIl17d*), *Mospd3* (*pancMospd3*) and *Tbc1d22a* (*pancTbc1d22a*) (Fig. 2A). First, we examined their expression patterns in the MII oocyte, sperm, and fertilized 1-cell and 2-cell embryo by RT-qPCR (Fig. 2B). We confirmed that all of these pancRNAs were expressed at the 2-cell stage, and found that the expression of *Mospd3* and *Tbc1d22a* mRNAs was also upregulated at the 2-cell stage (Fig. 2B, middle and right panels), whereas the expression of *Il17d* mRNA was first detected at the 4-cell stage (Fig. 2B, left panel). Thus, expression of the pancRNA preceded or occurred simultaneously with that of the mRNA at these loci during early embryogenesis.

Next, we analyzed whether the promoter methylation status reflects the gene activation/repression status. Since core promoter regions, which frequently show high CpG density, tend to be constitutively hypomethylated, and the flanking sequences with lower CpG density tend to be associated with developmental gene regulation (Meissner et al., 2008), we surveyed such developmentally regulated regions using publicly available MethylC-seq data of mouse germ cells and 2-cell embryos (Wang et al., 2014) at the three loci (supplementary material Fig. S6). Bisulfite sequencing indicated that this region in the *Il17d* promoter is considerably methylated at the MII oocyte, sperm and 1-cell stages (Fig. 2C). By contrast, this region became almost completely demethylated by the 2-cell stage, while the region located nearer the TSS was constitutively free of methylation, as expected from the MethylC-seq data (supplementary material Fig. S7). Similarly, the promoter regions of *Mospd3* and *Tbc1d22a* were methylated at the MII oocyte, sperm and 1-cell stages, and their DNA methylation levels decreased by the 2-cell stage. The concordance between the observed kinetics of expression of the pancRNAs and the changes in DNA demethylation raised the possibility that these pancRNAs mediate gene activation through epigenetic changes.

**Fig. 3.**
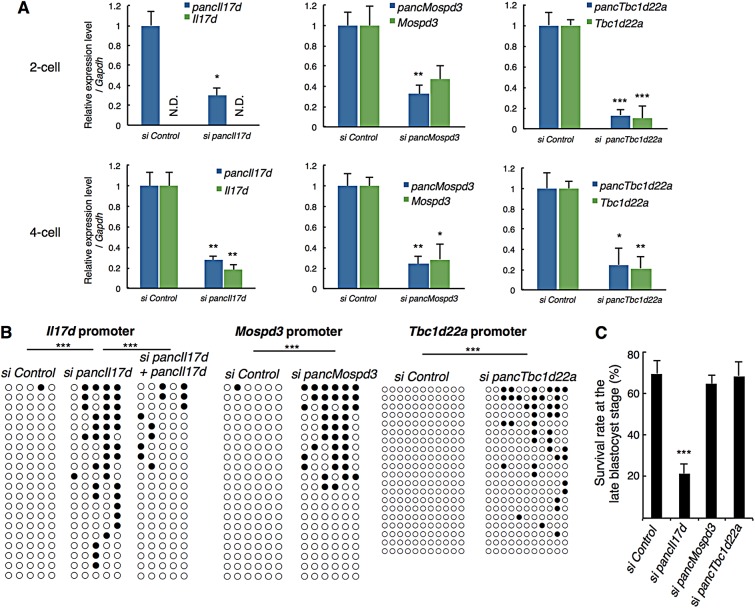
**Effect of knockdown of pancRNAs on partnered gene expression and on DNA methylation.** (A) Expression levels of the indicated pancRNAs and their genes measured by qPCR in siRNA-injected 2-cell and 4-cell embryos. *Il17d* expression was not detectable (N.D.) in 2-cell embryos. (B) DNA methylation levels of the corresponding promoters in siRNA-injected embryos. The regions analyzed are displayed in Fig. 1A. (C) Effect of pancRNA knockdown on blastocyst formation. Asterisks indicate significant differences compared with si Control samples. The numbers of embryos used for injection of si Control, si *pancIl17d*, si *pancMospd3* and si *pancTbc1d22a* siRNAs were 261, 251, 78 and 70, respectively. *P<0.05; **P<0.01; ***P<0.001. Error bars indicate s.e.m.

Since we previously found that pancRNAs could activate gene expression in rat differentiated cell lines (Tomikawa et al., 2011), we tested whether these developmentally expressed pancRNAs could be involved in the gene upregulation in early mouse embryos by knocking them down using siRNAs. We found that microinjection of siRNA for *pancIl17d*, *pancMospd3* or *pancTbc1d22a* into the pronucleus suppressed expression of the partner mRNA at the 2-cell and 4-cell stage, when partner expression normally begins (Fig. 3A), and this suppression was accompanied by a lack of decline in the methylation level in the respective promoter region (Fig. 3B; supplementary material Fig. S8). At the *Il17d* locus, this knockdown effect could be rescued by co-injection of the *pancIl17d* overexpression vector (Fig. 3B). Overexpressed *pancIl17d* might work as a sponge for the siRNA, and these pancRNAs might mediate acquisition of the hypomethylated status of the corresponding promoters and potentiate subsequent gene expression after fertilization.

### Developmental defect caused by *pancIl17d* knockdown during preimplantation stages

To investigate the effects of knocking down the above three pancRNAs on preimplantation development, we monitored the rate of successful blastocyst formation in the knockdown embryos (Fig. 3C). 69.6±6.4% of control siRNA-injected, 64.8±4.0% of *pancMospd3* knockdown and 68.6±6.6% of *pancTbc1d22a* knockdown embryos successfully developed to the late blastocyst stage *in vitro*. By contrast, only 21.5±4.7% of *pancIl17d* knockdown embryos reached the late blastocyst stage (Fig. 3C). This developmental defect was also produced using another siRNA for *pancIl17d* (supplementary material Fig. S9). Regarding *pancMospd3* knockdown, although we could not see clear effects in the blastocysts, we found a deficiency of hatching after extending the culture of such embryos. After 5 days, a significant fraction of the *pancMospd3* knockdown embryos did not hatch from the zona pellucida, whereas most of the control embryos hatched (supplementary material Fig. S10A,B). Similar results were observed in ESCs: *pancMospd3* knockdown resulted in a decreased number of cells compared with the control ESCs (supplementary material Fig. S10C).

**Fig. 4.**
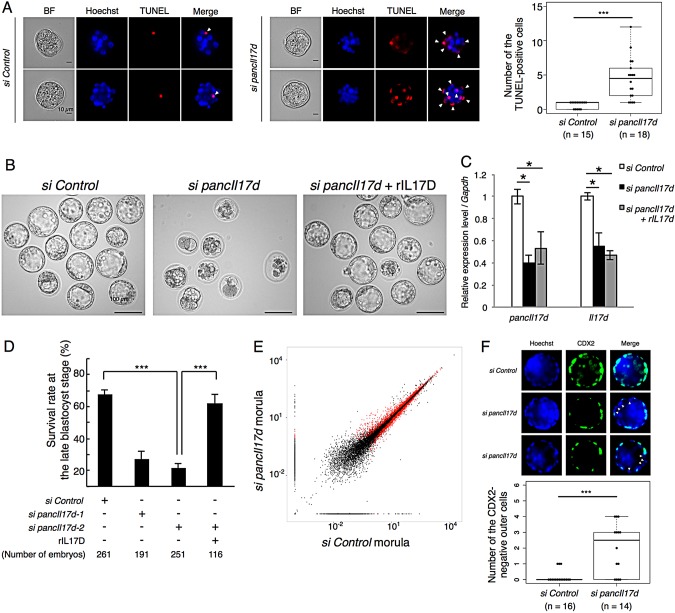
**Developmental defects induced by *pancIl17d* knockdown and rescue by addition of recombinant IL17D protein.** (A) TUNEL assay of morula embryos. Arrowheads indicate TUNEL-positive blastomeres. *pancIl17d* knockdown embryos showed increased TUNEL-positive cells. Two representative blastomeres are shown. To the right is a box plot of the number of TUNEL-positive cells in each embryo. (B) Morphology of late blastocysts. (C) Expression levels of *pancIl17d* and *Il17d* measured by qPCR in control, *pancIl17d* knockdown and rIL17D-supplemented *pancIl17d* knockdown morula embryos. (D) Survival rate of control and *pancIl17d* knockdown embryos at day 4.5 of *in vitro* culture. Two different siRNAs targeting *pancIl17d* were used. (E) Scatter plots of gene expression in control and *pancIl17d* knockdown morula embryos based on the RPKMs of RefSeq genes. Red dots indicate the genes that show statistically significant changes. (F) Immunostaining of CDX2 protein in control and *pancIl17d* knockdown late blastocyst. Arrowheads indicate CDX2-negative outer cells Beneath is a box plot of the number of CDX2-negative outer cells in each embryo *P<0.05; ***P<0.001. Error bars indicate s.e.m.

Since the effect of *pancIl17d* knockdown was drastic, we focused on investigating the roles of this pancRNA in embryonic development*.* Many *pancIl17d* knockdown embryos died between the 8-cell and early blastocyst stages. To establish whether cell death was enhanced in the *pancIl17d* knockdown embryos, we performed TdT-mediated dUTP nick-end labeling (TUNEL) staining of the *pancIl17d* knockdown embryos at the morula stage (Fig. 4A). Consistent with a previous report (Brison and Schultz, 1997), control embryos underwent little apoptosis during blastocyst formation. By contrast, *pancIl17d* knockdown embryos exhibited multiple TUNEL-positive blastomeres, suggesting that many *pancIl17d* knockdown embryos died by the blastocyst stage due to excessive apoptosis. It is noteworthy that the developmental capacity to form a blastocyst was restored when recombinant mouse IL17D protein (rIL17D) was added to the medium at the 4-cell stage, although the knockdown effect continued until the morula stage (Fig. 4B,C). The addition of rIL17D significantly increased the rate of success of blastocyst formation in *pancIl17d* knockdown embryos (from 21.5±2.7% to 62.1±5.9%; Fig. 4D). These results suggest that *pancIl17d* plays an important role in blastocyst formation by upregulating the partner gene.

In order to assess the role of *pancIl17d* in preimplantation embryos, we performed RNA-seq of *pancIl17d* knockdown morula embryos and compared the data with those for control siRNA-injected morula embryos (Fig. 4E). Gene ontology analysis revealed that apoptosis-related genes were enriched among the upregulated genes in the knockdown embryos (supplementary material Table S4). This is in accord with the observation that aberrant apoptosis is induced by *pancIl17d* knockdown, as shown in Fig. 4A. Interestingly, embryonic development-related genes were enriched among the downregulated genes. The second and third most highly expressed genes among the downregulated genes were *Nanog* and *Cdx2*, respectively, which encode transcription factors important for maintaining pluripotency and for the specification of cell lineages to generate trophectoderm, respectively (Chambers et al., 2003; Strumpf et al., 2005) (supplementary material Fig. S11). For example, the importance of pancRNAs for trophectoderm cell generation was supported by immunostaining of CDX2 of *pancIl17d* knockdown blastocysts, which showed that some outer blastomeres of *pancIl17d* knockdown embryos lost CDX2 expression (Fig. 4F). Therefore, *pancIl17d* seems important for the capacity to differentiate to generate trophectoderm cells.

### Impairment of *in vitro* colony formation from *pancIl17d* knockdown embryos

**Fig. 5.**
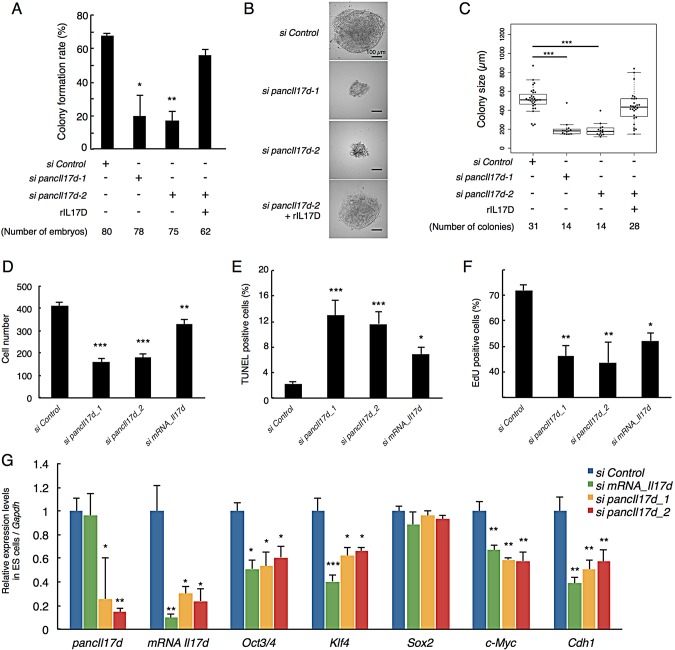
**Effect of *pancIl17d* knockdown on colony outgrowth from blastocysts and on ESC properties.** (A) Rate of colony outgrowth from knockdown and rescued blastocysts. Colonies growing after 10 days in culture were counted. (B) Representative images of colonies derived from siRNA-injected embryos. (C) Box plot of diameter of colonies derived from pancRNA knockdown blastocysts. (D) Number of ESCs 24 h after siRNA introduction by electroporation. (E) Proportion of apoptotic cells detected by TUNEL staining in knocked down ESCs after siRNA introduction. (F) Proportion of proliferating ESCs, as analyzed by EdU labeling. (G) Expression levels of *pancIl17d*, *Il17d* and pluripotency marker genes in ESCs, as detected by RT-qPCR. *Gapdh* was used as a control. The expression level in control-transfected ESCs was set as 1. Asterisks indicate significant differences compared with si Control samples. *P<0.05; **P<0.01; ***P<0.001. Error bars indicate s.e.m.

To further investigate the significance of *pancIl17d* for embryonic development, we plated the surviving *pancIl17d* knockdown blastocysts in medium containing mouse LIF and inhibitors for MEK1/2 (MAP2K1/2) and GSK3β (2i medium), conditions that are frequently utilized for the culture of ground-state ESCs, and harvested the cultures after 10 days. Whereas about 70% of the control siRNA-injected embryos produced ESC-like colonies on average, only 10-20% of the *pancIl17d* knockdown embryos did so (Fig. 5A). Even when *pancIl17d* knockdown embryos did produce colonies, they were significantly smaller than those derived from control siRNA-injected embryos (Fig. 5B,C), indicating that *pancIl17d* knockdown decreases the ability to form a colony. These knockdown-induced impairments were also rescued by the addition of rIL17D to the culture medium at the 4-cell stage, strongly suggesting that the effects of *pancIl17d* knockdown are mediated by the downregulation of *Il17d* gene expression.

We further investigated the effect of *pancIl17d* knockdown in ESCs. siRNA-induced *pancIl17d* knockdown resulted in a decrease in the number of ESCs compared with the control siRNA (Fig. 5D). When *pancIl17d* was knocked down in ESCs, TUNEL-positive cells were significantly increased compared with the control (Fig. 5E). These results indicated that the loss of *pancIl17d* led to apoptotic cell death also in ESCs. In parallel, we analyzed the proliferative ability of the *pancIl17d* knockdown ESCs by performing a 5-ethyl-2′-deoxyuridine (EdU) incorporation experiment. The number of EdU-positive cells was significantly decreased in the *pancIl17d* knockdown cells compared with the control cells (Fig. 5F). These inhibitory effects of pancRNA knockdown on the proliferation of ESCs were reproduced by mRNA knockdown (Fig. 5D-F). Taken together, these results indicate that the *pancIl17d-Il17d* pair forms a molecular axis that is necessary for both cell survival and proliferation.

We analyzed the expression of pluripotency marker genes, including *Oct3/4* (*Pou5f1*), by RT-qPCR. Knockdown of *pancIl17d* or of *Il17d* mRNA caused significant decreases in the expression levels of *Oct3/4*, *Klf4*, *c-Myc* and *Cdh1*, but not of *Sox2* (Fig. 5G). We also performed the embryoid body (EB) formation assay using shRNA-transfected ESCs (supplementary material Fig. S12). EB size was altered by transfection of shRNA for *pancIl17d*, accompanied by increased expression levels of *Otx1* and *Gata6*, which are marker genes for the ectodermal and endodermal lineages, respectively. This indicated that *pancIl17d* knockdown causes abnormal EB formation.

### Identification of the pathway triggering pancRNA-mediated gene upregulation

**Fig. 6.**
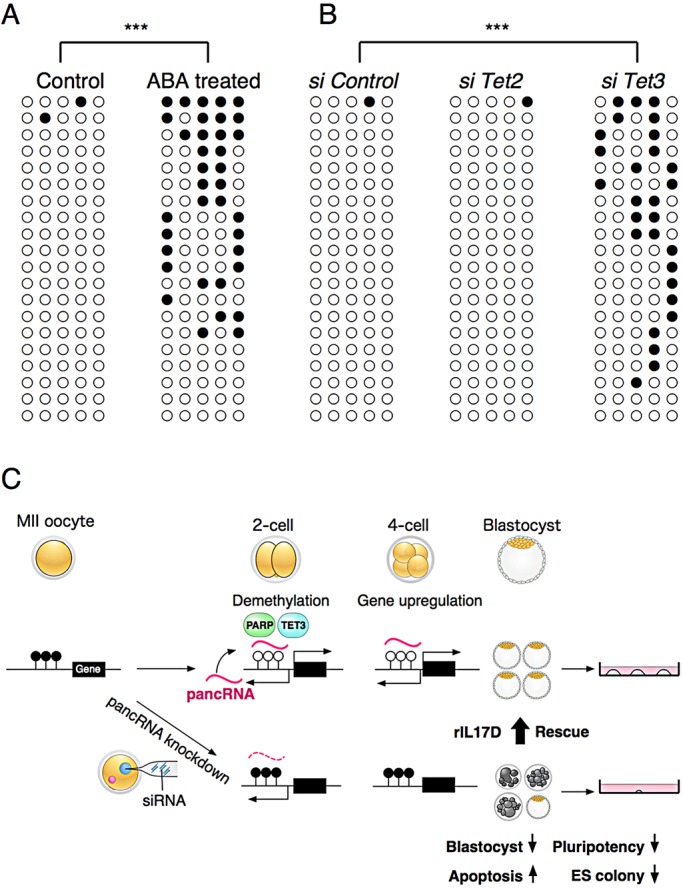
**Epigenetic changes accompanying zygotic pancRNA-mediated gene activation during early mouse development.** (A) DNA methylation status of ABA-treated 2-cell embryos. (B) DNA methylation status of *Tet2* or *Tet3* versus control siRNA-injected embryos. ***P<0.001. (C) A model for pancRNA-mediated gene activation in early mouse development. At the 2-cell stage, *pancIl17d* expression, together with TET3 and PARP, leads to establishment of the hypomethylated status at the *Il17d* promoter in a sequence-specific manner, and thus to *Il17d* mRNA expression starting from the 4-cell stage. When these steps are compromised, apoptosis increases and cell proliferation decreases, adversely affecting embryonic development.

A previous study showed that base excision repair (BER) components, including poly(ADP-ribose) polymerase (PARP), contribute to DNA demethylation in preimplantation embryos (Hajkova et al., 2010). Therefore, we added a PARP inhibitor, 3-aminobenzamide (ABA), to the embryo culture medium to clarify whether promoter demethylation requires the BER pathway. The addition of ABA resulted in inhibition of DNA demethylation of the *Il17d* promoter region at the 2-cell stage (Fig. 6A; supplementary material Fig. S13), leading to downregulation of the partner mRNA (supplementary material Fig. S14). However, the addition of ABA did not change pancRNA expression, suggesting that expression of *pancIl17d* itself is regulated independently of the BER pathway and DNA methylation.

Considering recent reports showing that ten-eleven translocation (TET) enzymes are among the key molecules triggering BER pathways (Kohli and Zhang, 2013; Teperek-Tkacz et al., 2011), we knocked down *Tet3*, which is abundantly expressed in early embryos, and *Tet2*, which shows lower expression than *Tet3* in preimplantation embryos (supplementary material Table S5). *Tet3* knockdown embryos showed significantly higher DNA methylation levels than control embryos. By contrast, knockdown of *Tet2* did not induce significant DNA methylation changes (Fig. 6B), suggesting that *Tet3*, but not *Tet2*, is required for DNA demethylation at the *Il17d* promoter. Fig. 6C summarizes pancRNA-mediated sequence-specific gene upregulation.

## DISCUSSION

The key molecules that enable sequence-specific gene activation to initiate embryonic development remain largely unknown. Here, we identified more than 1000 pancRNAs as candidates of such key molecules in early mouse embryos. To examine the function of the ZGA-associated pancRNA, we focused on three abundant pancRNAs: *pancIl17d*, *pancMospd3* and *pancTbc1d22a*. We found that these three pancRNAs had the ability to reprogram the epigenetic status of promoter regions for gene activation in a sequence-specific manner. We also proved that *pancIl17d* plays an essential role in early embryogenesis. Our study thus sheds light on novel mechanisms by which a fraction of zygotically activated lncRNAs enhance partner gene promoter activity for subsequent mouse embryogenesis.

### The effects of pancRNAs on gene regulation in many biological processes

In this study, the RNA-seq method was adapted for small-scale samples to yield RNA-seq data at a level comparable to that generated from large-scale samples (supplementary material Fig. S2). Indeed, pancRNAs were detected from more than 1000 promoter regions during ZGA (Fig. 1A). This is consistent with previous reports showing that thousands of pancRNAs are transcribed in terminally differentiated mouse tissues and ESCs (Sigova et al., 2013; Uesaka et al., 2014). Thus, a substantial number of pancRNAs seems to be expressed in various cell contexts, including totipotent stages, as we show here. Since pancRNAs and mRNAs exhibit coordinated expression changes not only in somatic cells but also in preimplantation embryos (Fig. 1D), pancRNAs might be commonly utilized for gene activation from the zygotic to the terminally differentiated stages. According to their partner genes, pancRNAs function in the regulation of many biological processes, a conclusion supported by our *pancIl17d* knockdown experiments.

### Sequence-specific transcriptional activation mediated by pancRNAs

There have been several reports on the molecular basis of lncRNA-mediated transcriptional regulation in *trans* [for a review see Fatica and Bozzoni (2014)]. For example, *HOTAIR* represents a set of lncRNAs that can influence dispersed genomic regions (Chu et al., 2011). By contrast, we now think that a single pancRNA acts to mediate corresponding gene activation in *cis*: knockdown of *pancIl17d*, *pancMospd3* or *pancTbc1d22a* resulted in downregulation of the partner gene, and this downregulation was accompanied by a hypermethylated status of the corresponding promoter regions (Fig. 3A,B). In addition, we found that *pancIl17d* expression preceded *Il17d* expression (Fig. 2B; supplementary material Fig. S15), also supporting the notion that the pancRNA epigenetically activates its partner gene in *cis*. However, we cannot completely exclude the possibility that a pancRNA affects expression of other genes in *trans*. Nonetheless, we believe that the *trans* effect, if any, on preimplantation development was relatively small, because several developmental defects caused by knocking down *pancIl17d* were rescued by addition of rIL17D protein. These findings strongly support the idea that a pancRNA specifically regulates expression of the gene with which it shares a bidirectional promoter region.

### The mechanism of pancRNA-triggered gene activation

One possible scenario is that gene activation-associated pancRNAs specify the genomic position for establishing an epigenetic status that is conducive to gene activation with TET3 and BER components (Fig. 6A,B), which are involved in genome-wide DNA demethylation (Branco et al., 2012; Hajkova et al., 2010). However, we do not yet know what factors initiate the expression of gene activation-associated pancRNAs. In this study, we tried to identify such factors and found strand-specific enrichment of a CT-rich motif in a set of zygotic pancRNA-partnered genes (Fig. 1F). The TSSs of the pancRNA-partnered genes provide the switching points for the observed asymmetric distribution of the CT-rich motif (supplementary material Fig. S5). Considering the divergent transcription of mRNA and pancRNA, the distribution pattern of this CT-rich motif seems to be preferentially located upstream of both pancRNAs and mRNAs. This raises the possibility that the coordinated expression of pancRNAs and mRNAs is regulated by similar machineries. This hypothesis is supported by the fact that the expression of 426 out of 568 pancRNAs increased at the 2-cell stage concomitantly with the increase in corresponding mRNA expression (supplementary material Fig. S3). However, the factor that binds to this CT-rich motif remains to be identified; indeed, the CT-rich motif is present upstream of *pancIl17d* and *pancTbc1d22a*, but not *pancMospd3*, and therefore information on additional sequence motifs and their binding factors will be needed to clarify the driving force that reprograms the chromatin structure in conjunction with pancRNA activation.

Although the pancRNA expression change seems to coincide with the DNA methylation change during preimplantation development, the effect on DNA methylation might be exerted indirectly. For example, DNA methylation and histone modification work together in gene silencing, and pancRNAs might initially affect some epigenetic/transcriptional environmental condition, such as histone modification status, leading to the DNA demethylation. Further advances in histone modification analysis techniques will enable us to dissect the exact kinetics of epigenetic changes triggered by pancRNA expression and thus aide in the identification of the molecular complex(es) that functions with pancRNA for sequence-specific gene activation.

### Developmental roles of pancRNAs

It is clear that the *pancIl17d-Il17d* pair performs some functions at the preimplantation stage. We speculate that some of the other upregulated pancRNA/gene pairs affect embryonic development. Several lncRNAs have also been shown to be involved in mouse postimplantation development [for a review see Fatica and Bozzoni (2014)]. For example, knockout mice of the lncRNA *Fendrr*, which is derived from the promoter region of *Foxf1*, die around embryonic day 14 due to impairment of heart development. Although knockdown of *pancMospd3* did not cause any detectable developmental defects in blastocyst formation (Fig. 3C), it must function thereafter, since mice lacking the *Mospd3* gene display neonatal lethality with defects of heart development (Pall et al., 2004). This notion is supported by our data showing that *pancMospd3* knockdown caused failure of hatching from the zona pellucida (supplementary material Fig. S10).

Interestingly, cell death-related genes were enriched among co-upregulated pancRNA-partnered genes (supplementary material Tables S1 and S2). These include *Bag6*, *Pdcd2*, *Map3k7* and *Fadd*, which are essential for embryonic development (Fabian et al., 2005; Jadrich et al., 2006; Mu et al., 2010; Yeh et al., 1998). For example, *Bag6* knockout mice die with defects of kidney, lung and brain formation as a result of dysregulation of apoptosis and cell proliferation (Fabian et al., 2005). In accord with this, our *pancBag6* knockdown experiment showed increased cell death among ESCs (supplementary material Fig. S16). Therefore, pancRNAs seem to be produced at a significant number of gene promoters that should be regulated according to the developmental context.

This raises the intriguing question of why pancRNAs are employed within the developmental gene regulation network. One possibility is that pancRNAs have been adopted to increase the complexity of the regulatory network system. The novel layer of transcriptional regulation imposed by the acquisition of pancRNAs might have contributed to generating numerous varieties of gene expression patterns during development (Imamura et al., 2014). Recently, it has been reported that lncRNAs, including pancRNAs, are frequently regulated by developmentally important factors, such as homeobox proteins, (Necsulea et al., 2014), supporting our idea that pancRNAs acts together with other regulatory factors for complex and orchestrated developmental gene regulation.

The knockdown of *pancTbc1d22a* and *pancBag6* did not cause marked defects in preimplantation development. As described above, pancRNAs function in the regulation of many biological processes according to their partner genes. In fact, *Tbc1d22a* belongs to the TBCK gene family, whose members are thought to act as GTPase-activating proteins and to influence cell proliferation through mTOR signaling (Alexander et al., 2013; Liu et al., 2013). Thus, although *Tbc1d22a* might have a role during development, it is possible that paralogs of *Tbc1d22a* might compensate for the knockdown effect. The same might be true for *pancBag6*, the knockdown of which resulted in only a slight decrease in ESC number (supplementary material Fig. S16).

### Conclusion

We conclude that gene activation-associated pancRNA provides a new layer of epigenetic regulation during mammalian development.

## MATERIALS AND METHODS

### Preparation of oocytes, embryos, sperm and ESCs

MII oocytes were obtained from the oviducts of 7- to 8-week-old F1 mice (C57BL/6×C3H) induced to superovulate by intraperitoneal injection of 5 IU of pregnant mare serum gonadotropin (Asuka), followed 48 h later by injection of 5 IU of human chorionic gonadotropin (hCG, Asuka). Embryos were obtained after mating the superovulated females with F1 males. Oocytes and zygotes were recovered in M2 medium (Sigma) 17 h after hCG injection, and then, following removal of cumulus cells with 0.03% hyaluronidase (Sigma), they were either subjected to direct methylation analysis and RNA analysis, or cultured in M16 medium (Sigma) at 37°C under 5% CO_2_/air for the collection of fertilized embryos. Sperm were obtained from F1 male epididymis, and motile sperm of good quality were selected by the direct swim-up method (Younglai et al., 2001). The embryos were treated with ABA (Sigma) as previously described (Imamura et al., 2004a). Blastocysts were plated in N2 medium containing B27 (Invitrogen), 2-mercaptoethanol (Wako), GlutaMAX-I (Gibco), bovine serum albumin fraction V (Sigma), LIF (Millipore), PD0325901 (Sigma) and CHIR99021 (Axon) (2i medium) (Ying et al., 2008) and cultured for 10 days. ESC-like colonies were processed for immunohistochemistry. ESCs were cultured on a 0.1% gelatin-coated dish in a 37°C incubator under 5% CO_2_/air, and propagated by trypsinizing and replating every 2 or 3 days. EB formation and *in vitro* hatching are described in the supplementary methods.

### Directional RNA-seq library preparation

Total RNA and poly(A)^+^ RNA were extracted from pools, each of which contained 100 MII oocytes, 2-cell embryos (C57/B6×ICR), control siRNA-injected morula embryos or *pancIl17d* knockdown morula embryos, using the Dynabeads mRNA DIRECT Micro Kit (Invitrogen). Four replicates were made for directional RNA-seq library construction using the NEBNext Ultra Directional RNA Library Prep Kit for Illumina (NEB). In this library preparation, cDNAs were enriched by 15-cycle PCR. Illumina HiSeq 2000 was used to perform 50 bp single-end sequencing according to the manufacturer's instructions. RNA-seq data have been deposited in the DDBJ Sequence Read Archive (DRA) under accession number DRA002400.

### Data mining

Sequencing reads obtained from our directional RNA-seq (DRA:DRA002400) and publicly available data [NCBI Sequence Read Archive (SRA)] for ESCs (SRA:SRR315596) were assessed with the FASTX tool kit (Patel and Jain, 2012) to remove short (<20 bp) and low quality (quality score <20) reads, followed by trimming of the adaptor sequence.

Preprocessed reads were mapped to the mouse mm10 genome using TopHat2/Bowtie2 (Kim et al., 2013). Cufflinks and Cuffdiff (Trapnell et al., 2012) were used for the reads per kb of exon model per million mapped reads (RPKM) calculation and differential expression analyses. For pancRNA quantification, we counted only reads that mapped to the antisense sequences of the promoter regions (−1000 to −1 bp from the TSS) of genes, because pancRNAs corresponding to antisense sequences of the promoter regions show the potential to increase mRNA production (Tomikawa et al., 2011; Uesaka et al., 2014). If a promoter region overlapped with another RefSeq gene, the promoter was excluded from the dataset to avoid contamination of the pancRNA pool by protein-coding genes. Hierarchical clustering of sequenced samples based on gene expression levels was drawn using the cummeRbund package (http://rgm3.lab.nig.ac.jp/RGM/R_package_list). For motif searches within the promoter sequences, ≥10-fold upregulated pancRNAs in 2-cell embryos were selected by comparing their levels with those in MII oocytes. The −200 to −1 bp sequences (relative to the TSS) of 370 corresponding mRNAs were examined using rGADEM, one of the Bioconductor packages (Gentleman et al., 2004). To verify the presence of a motif in the pancRNA-partnered gene loci, we further extracted and counted genes that possessed or lacked sequences showing 90% or more identity to the candidate motif using the matchPWM program in the Biostrings package (Pages et al., 2013). pancRNA-mRNA sets subjected to the experiments described below were selected based on the following criteria: RPKM <0.5 in MII oocytes, RPKM >1 in 2-cell embryos and ESCs (supplementary material Table S3).

### PCR detection of pancRNA and mRNA

To quantify the pancRNA and mRNA expression levels in the embryos, we purified total RNAs from sperm, oocytes, and fertilized 1-cell (corresponding to 30 h after hCG injection), 2-cell (44 h) and 4-cell (54 h) embryos using the Dynabeads mRNA DIRECT Micro Kit and subjected them to reverse transcription. For ESCs, 3 µg total RNA that had been extracted using TRIzol (Invitrogen) was utilized for reverse transcription with SuperScript III (Invitrogen) reverse transcriptase. The synthesized cDNAs were subjected to qPCR using the KAPA SYBR Fast qPCR Kit (KAPA Biosystems). The primers used in these analyses are listed in supplementary material Table S6. *Gapdh* was used as an internal control.

### Bisulfite sequencing

To determine the DNA methylation profiles of the *Il17d*, *Mospd3* and *Tbc1d22a* promoter regions, sample pools consisting of genomic DNA from 20-50 oocytes or embryos were subjected to the bisulfite reaction using the MethylCode Kit (Invitrogen) according to the manufacturer's instructions. Each bisulfite-treated genome was amplified using AmpliTaq Gold 360 Master Mix (Life Technologies) or EpiTaq HS (TaKaRa) and the specific primers listed in supplementary material Table S6. In order to avoid PCR bias, we subcloned more than five PCR bands as previously described (Imamura et al., 2005), and performed bisulfite sequencing of more than 20 of the resulting subclones, for each sample. Visualization of MethylC-seq data is described in the supplementary methods.

### pancRNA knockdown and overexpression experiments

We microinjected 5-10 pl of 2 µM siRNA that targeted pancRNA of *Il17d*, *Mospd3* or *Tbc1d22a* (supplementary material Table S7), together with 5 ng/μl N2-EGFP vector (Clontech), into the pronuclei of fertilized embryos 21 h after hCG injection. In a rescue experiment of siRNA knockdown, 5 ng/μl *pancIl17d* overexpression construct [−706 bp to −418 bp relative to the TSS of *Il17d* in pRC/CMV (Invitrogen)] was simultaneously microinjected into the pronuclei. In the case of siRNAs that targeted *Tet2* and *Tet3* mRNAs, 5-10 pl of each siRNA at 50 µM was injected into the cytoplasm of embryos 14 h after hCG injection. As a negative control for siRNA experiments, we used the MISSION siRNA universal negative control (Sigma). siRNA-injected embryos were used for DNA methylation and RNA analyses. To look for possible morphological changes, *in vitro* culture was continued for 3 more days. In some cases, recombinant IL17D (R&D Systems) was added to a final concentration of 100 ng/ml at the 4-cell stage.

For knockdown experiments, ESCs were transfected with each siRNA (100 nM final concentration) as listed in supplementary material Table S7, together with pEGFP-N2 vector (Clontech), by electroporation with the Neon Transfection System (Invitrogen). At 24 h after transfection, cells were used for TUNEL assay, EdU labeling assay or RT-qPCR. For longer duration knockdown experiments in ESCs, pLLX-shRNA expression vectors, which were generously provided by Drs Z. Zhou and M. E. Greenberg (Lois et al., 2002; Zhou et al., 2006) and were modified to express GFP together with a puromycin resistance gene under the ubiquitin C promoter, were prepared as listed in supplementary material Table S7. Human embryonic kidney cells were used as producers of lentiviruses that contained the modified pLLX-shRNA expression vectors. After 2 days of infection, ESCs were selected with puromycin for 3 days to check their phenotypes.

### Cell staining

Immunohistochemistry was performed as follows: fixation with 4% PFA for 20 min at room temperature; washing twice in PBS; permeabilization and blocking in blocking buffer (0.1% Triton X-100 and 3% FBS in PBS) for 1 h at room temperature; overnight incubation with primary antibodies diluted 1/500 in blocking solution; washing three times in PBS; incubation with Hoechst 33258 (Nacalai Tesque) and secondary antibody diluted 1/500 in blocking solution for 1 h in the dark at room temperature; and washing three times in PBS. Imaging was performed with a Leica AF6000 microscope. The primary antibody mouse anti-CDX2 (MU392A-UC, BioGenex) was used for immunostaining. CF488A donkey anti-mouse IgG (Biotium) secondary antibody was used to visualize signals. For the TUNEL assay, cells were stained with TMR Red using the *In Situ* Cell Death Detection Kit (Roche) according to the manufacturer's instructions. For the EdU assay, EdU of the Click-iT EdU Imaging Kit (Invitrogen) was added to the ESC culture medium by exchanging half the medium and culturing for 4 h; the cells were then fixed with 4% PFA, permeabilized with 0.1% Triton X-100 in PBS, and stained with 1× Click-iT Reaction Buffer and Hoechst 33258.

### Statistical analysis

All data are reported as the mean±s.e.m. Student's *t*-test was used for comparisons between two groups. Unless there is a specific statement about the number of replicates, three replicates were analyzed for each experiment. Tukey's multiple comparison test was used for comparisons among three or more groups. The Mann–Whitney *U*-test was used to compare DNA methylation levels among samples.

## Supplementary Material

Supplementary Material
