# Supplementary material for: Gene activation-associated long noncoding RNAs function in mouse preimplantation development
Source: Development. 2015 Mar 1;142(5):910–20. doi: 10.1242/dev.116996 (PMC4352986; doi:10.1242/dev.116996)
Supplement: Supplementary Material [file supp_142_5_910__index.html]

Supplementary Material 

# Gene activation-associated long noncoding RNAs function in mouse preimplantation development

## DEV116996 Supplementary Material

**Files in this Data Supplement:**

- Supplementary Material
