# Supplementary material for: Gene activation-associated long noncoding RNAs function in mouse preimplantation development
Source: Development. 2015 Mar 1;142(5):910–20. doi: 10.1242/dev.116996 (PMC4352986; doi:10.1242/dev.116996)
Supplement: Supplementary Material [file supp_dev.116996_DEV116996supp.pdf]

Supplementary materials and methods

Embryoid body formation

Ten thousand ES cells were put on a Lipidure-Coat 96-well U-bottom plate (Thermo Scientific) and were cultured in the Alpha modification of Eagle's MEM (Gibco) supplemented with 10% FBS for 0, 3 or 6 days, and then 3 μg of total RNA that had been extracted using TRIzol (Invitrogen) was utilized for reverse transcription with SuperScript III (Invitrogen). The synthesized cDNAs were subjected to qPCR using a KAPA SYBR Fast qPCR Kit (KAPA Biosystems). The primers used in these analyses are listed in supplementary material Table S6. *Gapdh* was used as an internal control.

Visualization of the MethylC-seq

Mapped MethylC-seq data of oocytes, sperm, 2-cell embryos were obtained from GSE56697 and visualized by using GenomeJack, a genome viewer software (http://genomejack.net/english/index.html).

***In vitro* hatching**

We injected the siRNA for control, *pancTbc1d22a*-knockdown or panc*Mospd3*-knockdown into fertilized 1-cell stage embryos and cultured them in M16 medium (Sigma) at 37°C under 5% CO_2/air until the blastocyst stage. The blastocysts were cultured in 2i medium at 37°C under 5% CO_2/air for 5 days, and the rate of hatching from zona pellucida was calculated.

Supplementary figure legends

Fig. S1. Assessment of directional RNA-seq sample reads. (A) Scatter plots of gene expression in all sequenced samples. Each sample category consisted of four replicates each of MII oocytes (MII_1 to MII_4) and 2-cell embryos (F2_1 to F2_4). RPKMs of the RefSeq genes are plotted. (B) Hierarchical clustering of sequenced samples based on the gene expression levels. Jensen-Shannon distance was used for drawing the dendrogram, which indicates that our directional RNA-seq data clearly discriminate MII oocytes and 2-cell embryos.

Fig. S2. Density plots of RNA-seq reads mapped to the RefSeq genes. For MII oocytes and 2-cell embryos, we compared the reads reported by Park *et al*., 2013 (A) with our

datasets (B). Our data previously reported in Uesaka *et al*., 2014 are shown as an example of an adult tissue sample (C).

Fig. S3. Number of upregulated pancRNAs and mRNAs in 2-cell embryos. The Venn diagram shows the number of pancRNAs (568 in total) and mRNAs (520 in total) upregulated two-fold or more at the 2-cell stage. Note that the expression levels of most of the mRNAs and their corresponding pancRNAs are co-regulated.

Fig. S4. The existence of CpG island (CpGi) and CT-rich motif in pancRNA-partnered genes. (A) The occurrence of the CpGi within the pancRNA-partnered genes. (B) The occurrence of the CT-rich motif within the CpGi-genes. (C) The occurrence of the CT-rich motif within the pancRNA-partnered genes.

Fig. S5. Schematic representation of genomic features in pancRNA-lacking and pancRNA-partnered genes.

Fig. S6. The representation of MethylC-seq data (Wang et al., 2014) and our RNA-seq data at *Il17d*, *Mospd3* and *Tbc1d22a* loci. Amplified regions are shaded.

Fig. S7. The DNA methylation status of the region around the TSS of *Il17d* before and after fertilization. Thick horizontal lines denote the regions analyzed by bisulfite sequencing. Primer positions are numbered relative to the TSS of *Il17d*.

Fig. S8. The DNA methylation status of the region around the TSS of *Il17d* in *pancIl17d*-knockdown 2-cell embryos.

Fig. S9. Survival rate of control and *pancIl17d*-knockdown embryos at various developmental stages. For all knockdown experiments, siRNA for pancRNA was injected at the pronuclear stage. Early and late blastocysts were collected from cultures at times corresponding to 69 h and 88 h after hCG injection, respectively. Note that knockdown of *pancIl17d* reduced the viability of embryos by the early blastocyst stage. Asterisks indicate significant differences compared with *si Control* samples. ***, $p < 0.001$.

Fig. S10. Developmental effect of *pancMospd3*-knockdown. (A) Morphology of the si Control- and si *pancMospd3*-injected embryos 5 days after outgrowth *in vitro*. Arrows indicate the zona pellucida. Numbers of embryos used for the experiment of si Control, si *pancMospd3* and si *pancTbc1d22a* siRNAs were 177, 106 and 84, respectively. (B) The rate of hatching from zona pellucida. (C) The number of *pancMospd3*-knockdown ES cells. Cell count was performed 3 days after the passage of 1×10^5 cells.

Fig. S11. Relative expression levels of down-regulated and up-regulated top-3-ranked genes in *pancIl17d*-knockdown morula. Blue and red bars indicate the RPKM values of Control and *pancIl17d*-knockdown morula, respectively.

Fig. S12. Effect on the embryoid body (EB) formation in *pancIl17d*-knockdown ES cells. (A) Experimental scheme for shRNA-based *pancIl17d* knockdown in ES cells. Horizontal line indicates the time course of experiment. After the infection of shRNA for control and *pancIl17d* vectors, cells were selected for 5 days and utilized for embryoid body formation and qPCR analysis. (B) Representative control and

pancIl17d-knockdown EBs. (C) The diameters of EBs. (D) The knockdown efficiency of *pancIl17d* and *Il17d* at day 0. (E) Relative expression changes of marker genes during EB formation.

Fig. S13. The DNA methylation status of the region around the TSS of *Il17d* in *Tet3*-knockdown and ABA-treated 2-cell embryos.

Fig. S14. The relative expression levels of *Il17d* and *pancIl17d* in ABA-treated and control embryos.

Fig. S15. A representation of RNA-seq data of the *Il17d* locus. Blue and red signals indicate the expression levels of pancRNA and mRNA, respectively

Fig. S16. Developmental effect of *pancBag6*-knockdown. The number of *pancBag6*-knockdown ES cells. Cell counting was performed 3 days after the passage of $1x10^5$ cells.

Hamazaki_Fig. S1

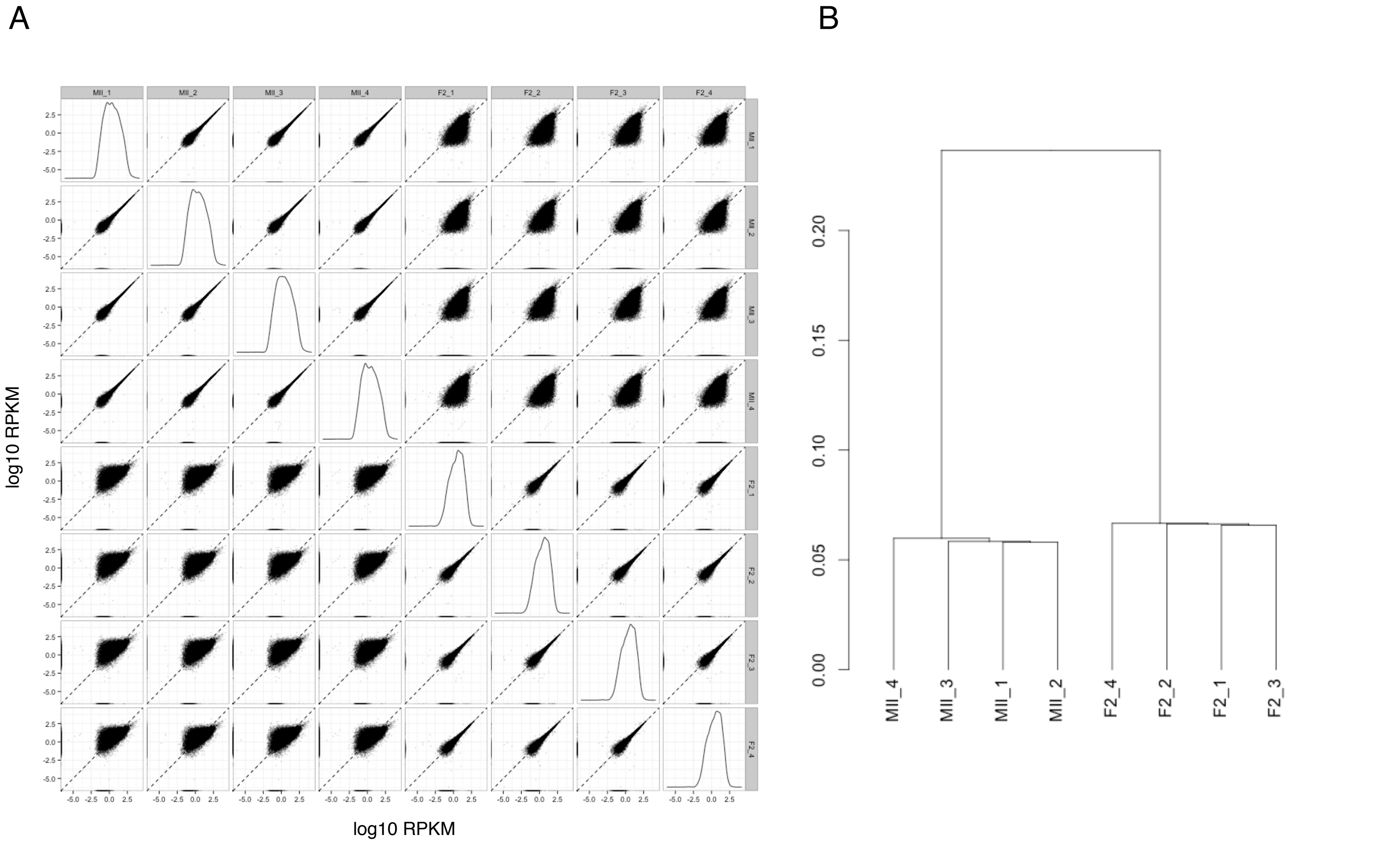

Hamazaki_Fig. S2

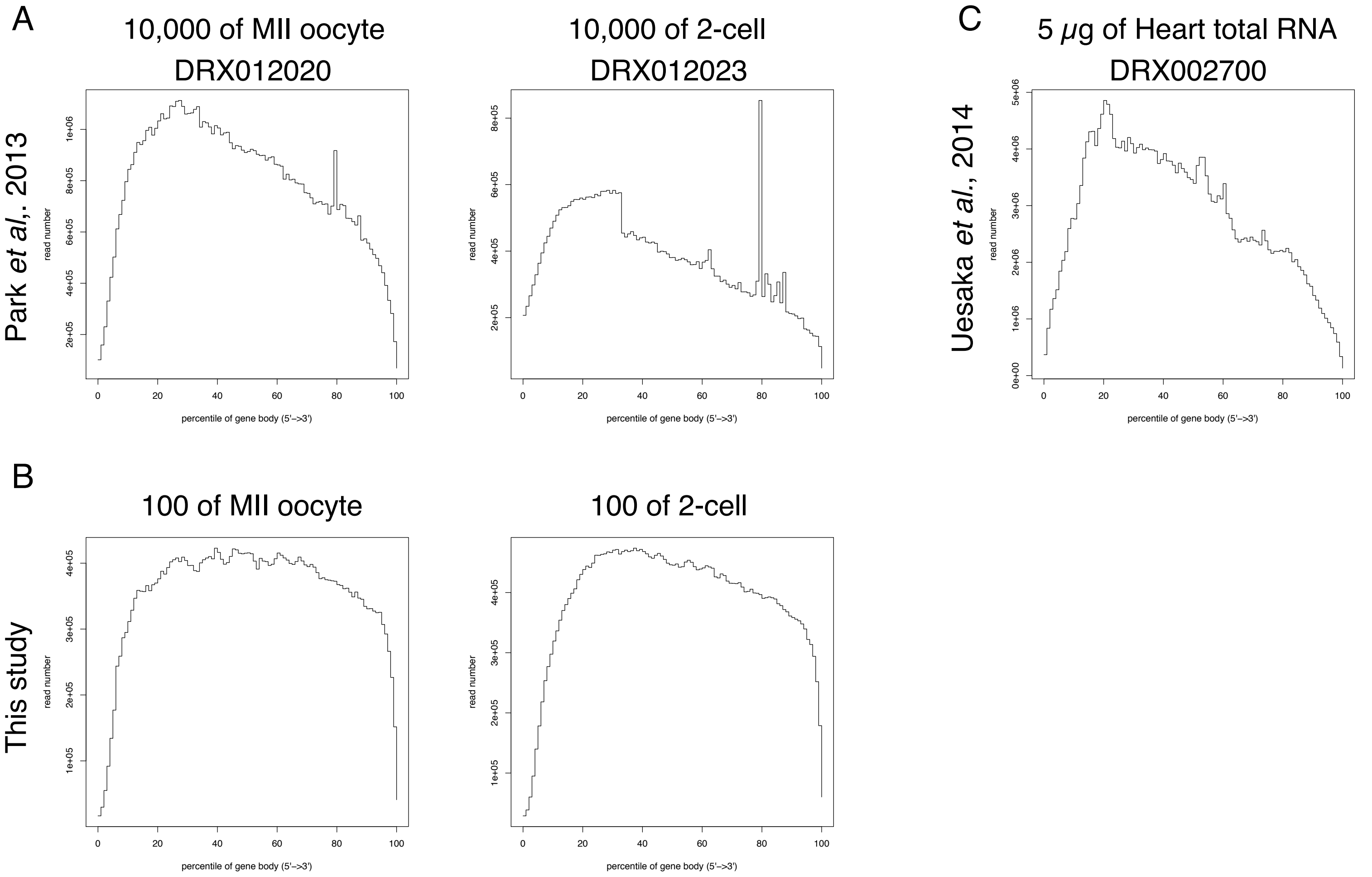

Hamazaki_Fig. S3

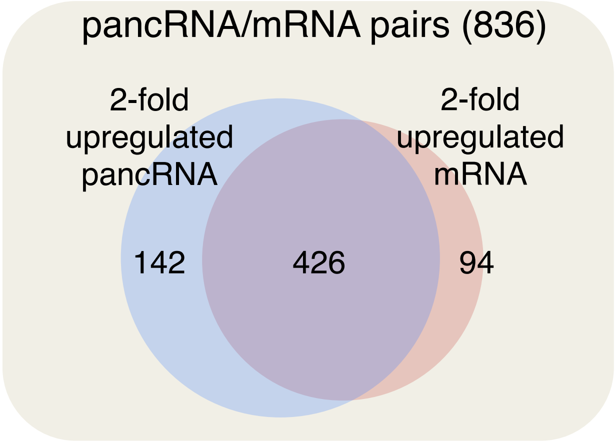

Hamazaki_Fig. S4

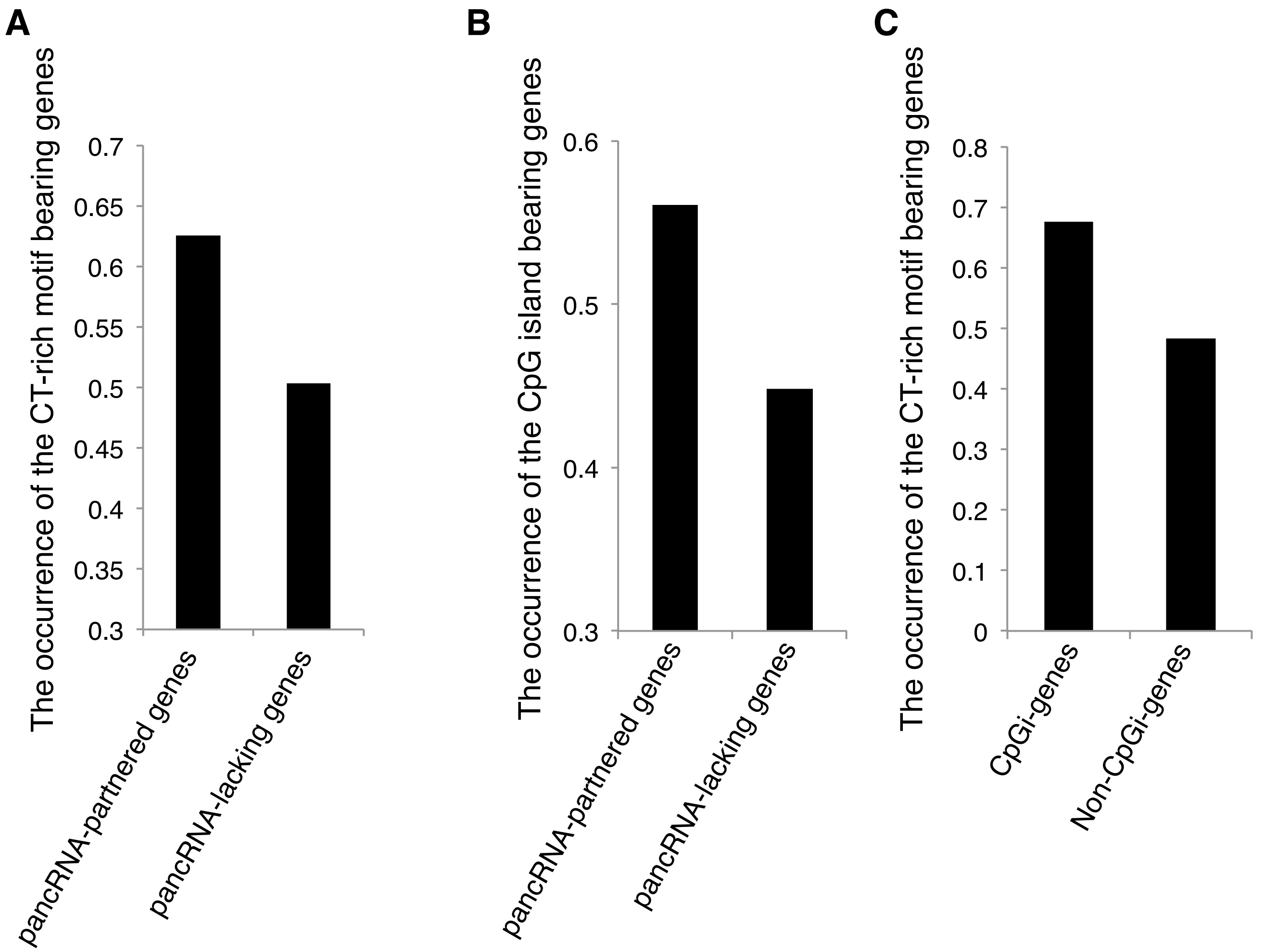

Development 142: doi:10.1242/dev.116996: Supplementary Material

Hamazaki_Fig. S5

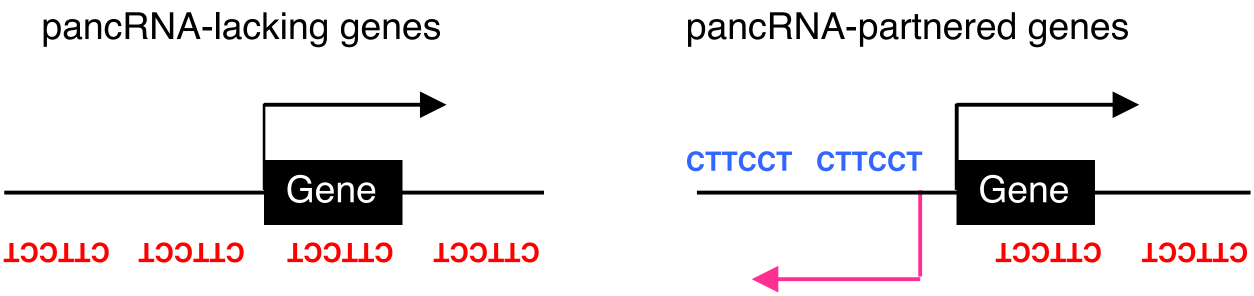

Hamazaki_Fig. S6

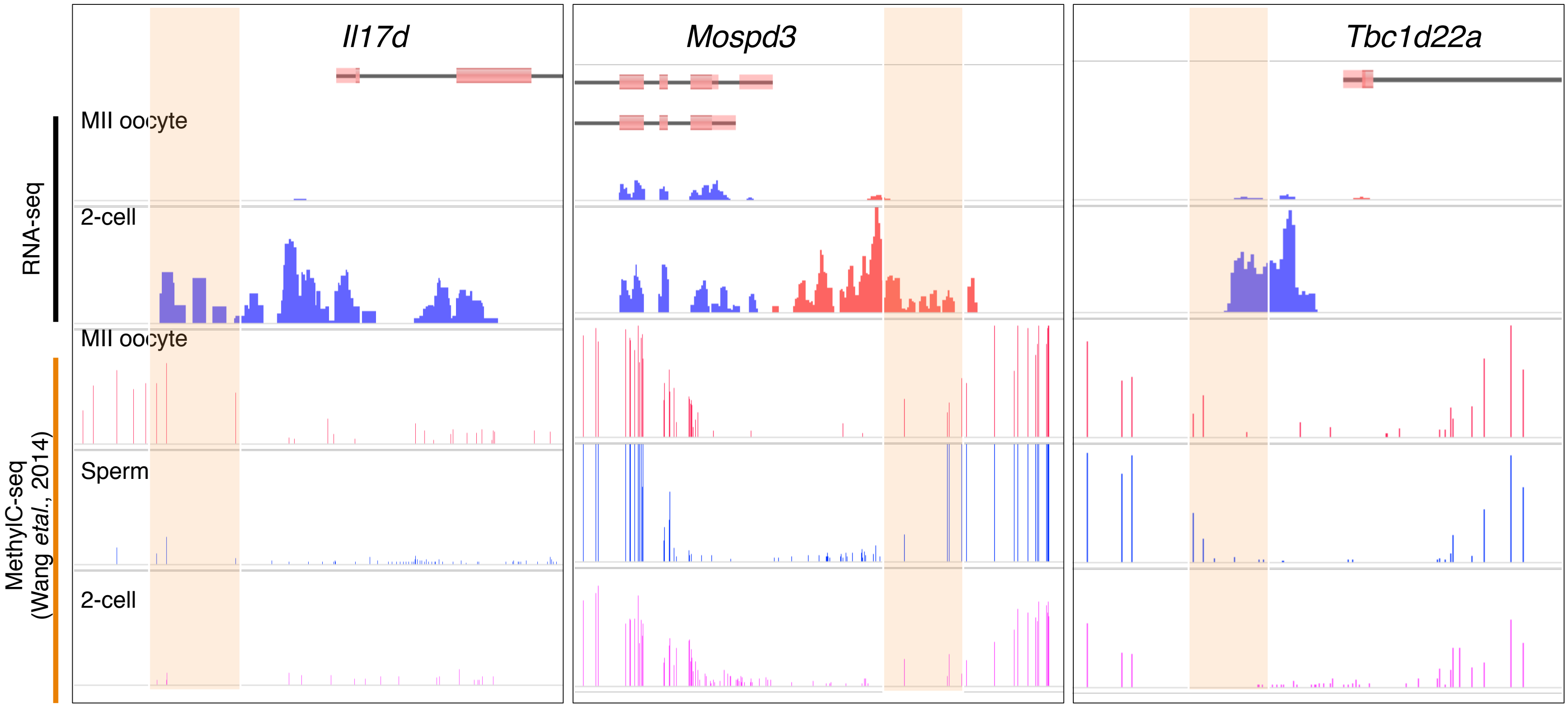

Hamazaki_Fig. S7

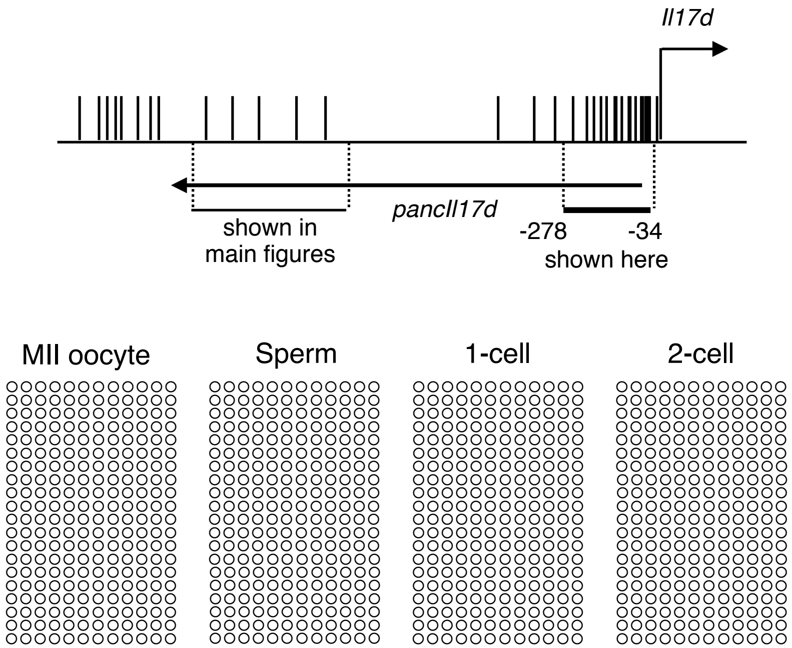

Hamazaki_Fig. S8

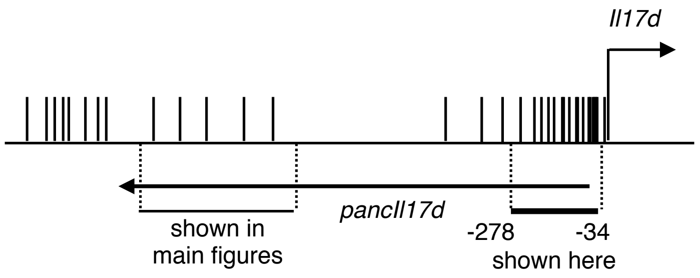

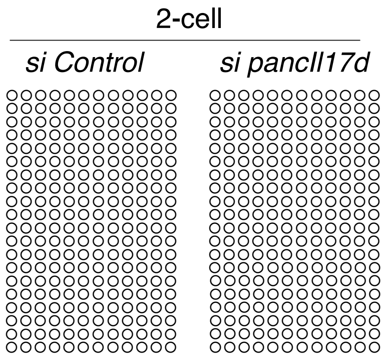

Hamazaki_Fig. S9

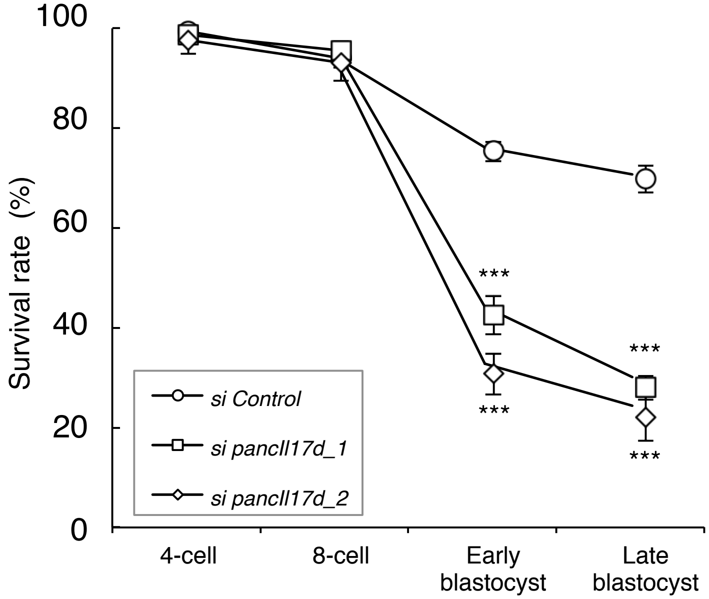

Hamazaki_Fig. S10

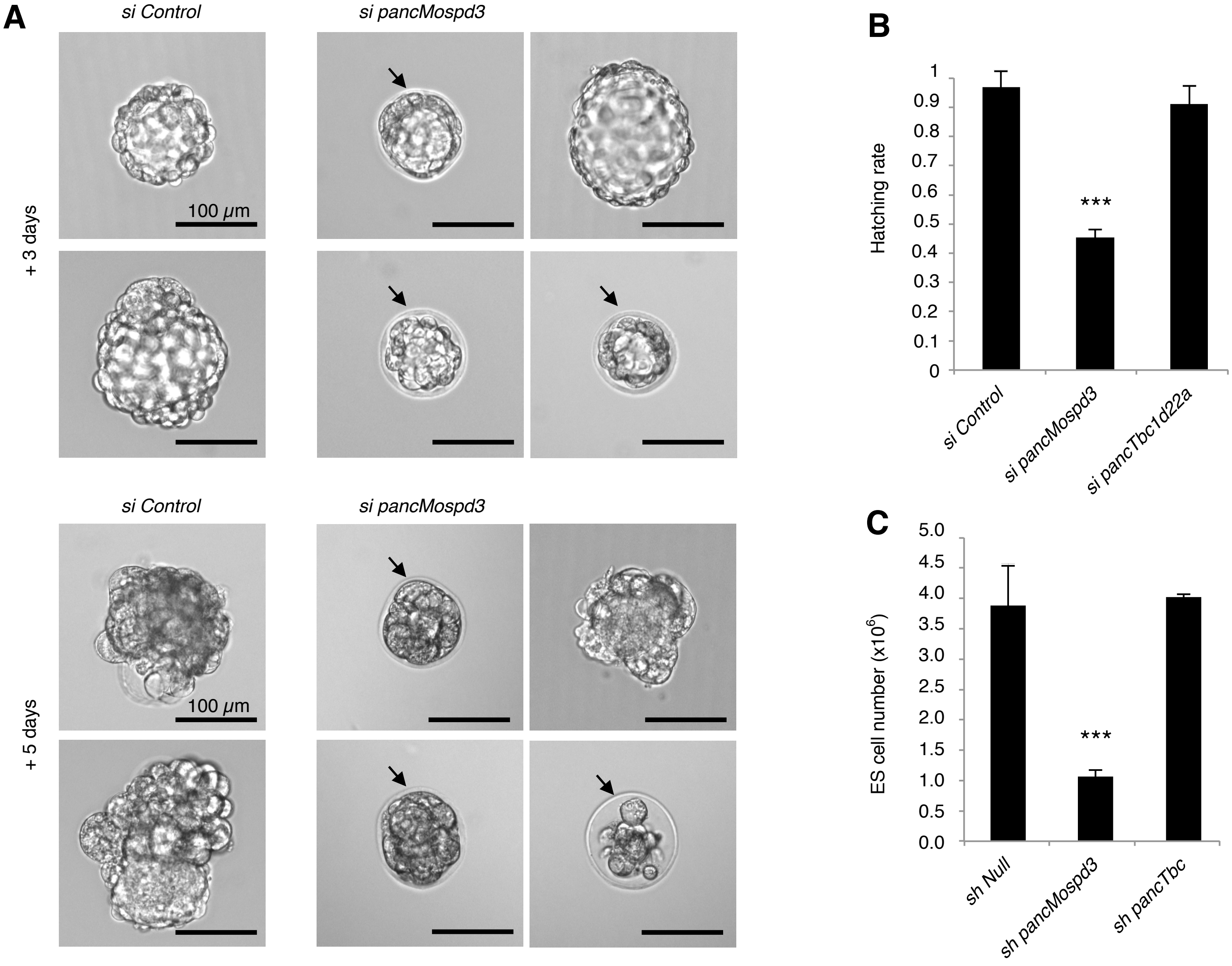

Hamazaki_Fig. S11

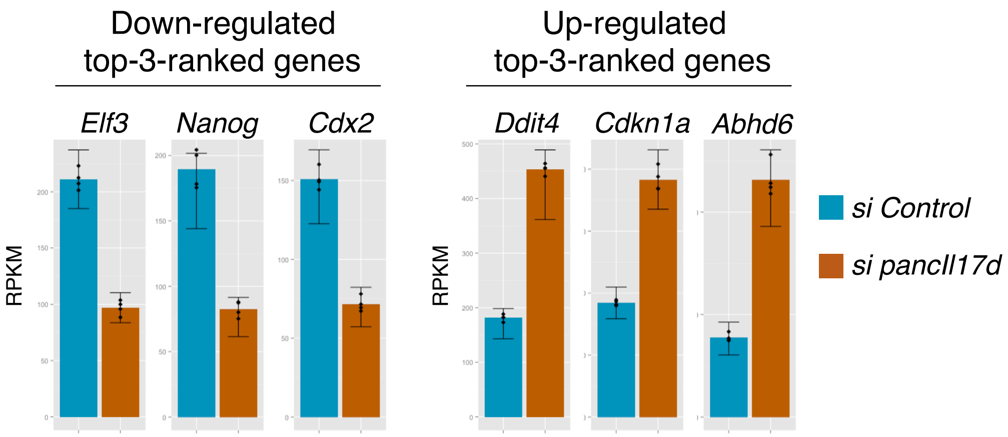

Development 142: doi:10.1242/dev.116996: Supplementary Material

Hamazaki_Fig. S12

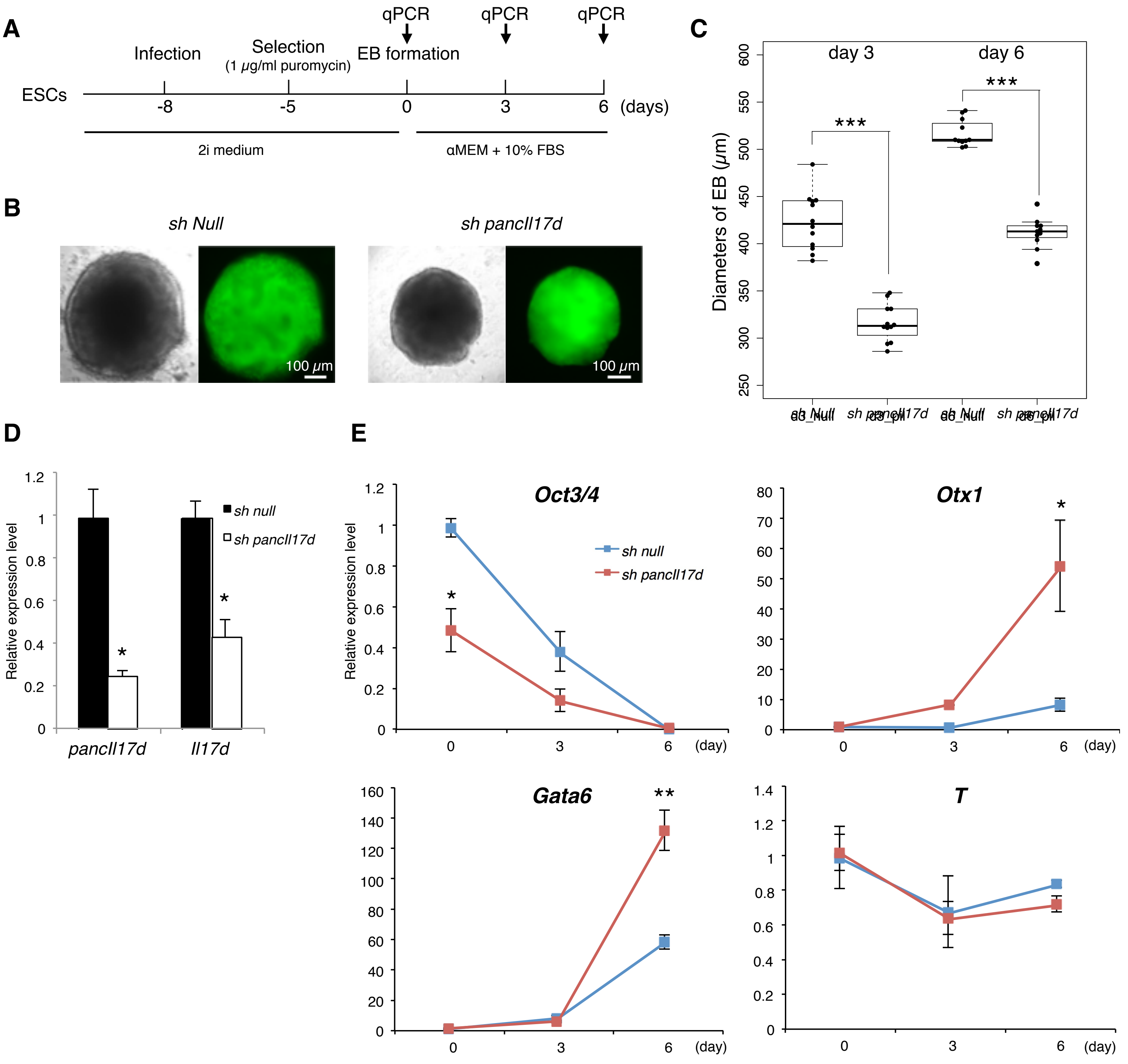

Hamazaki_Fig. S13

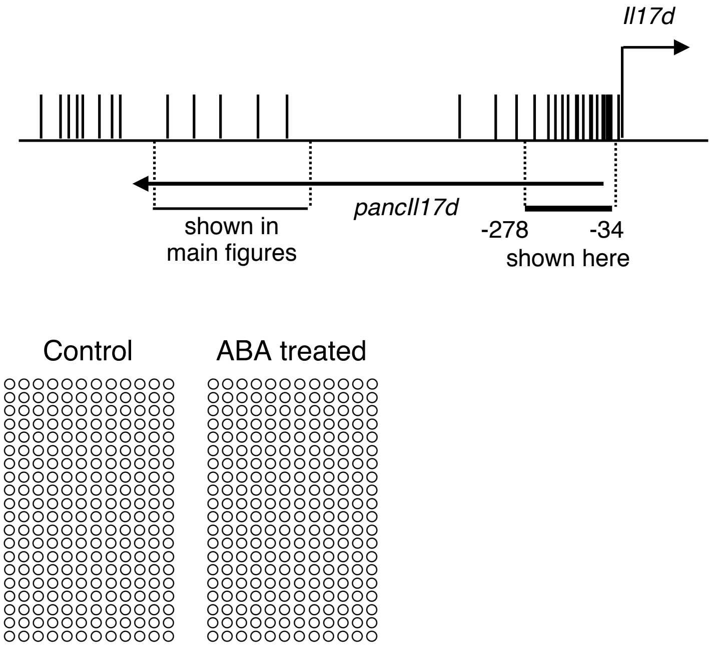

Hamazaki_Fig. S14

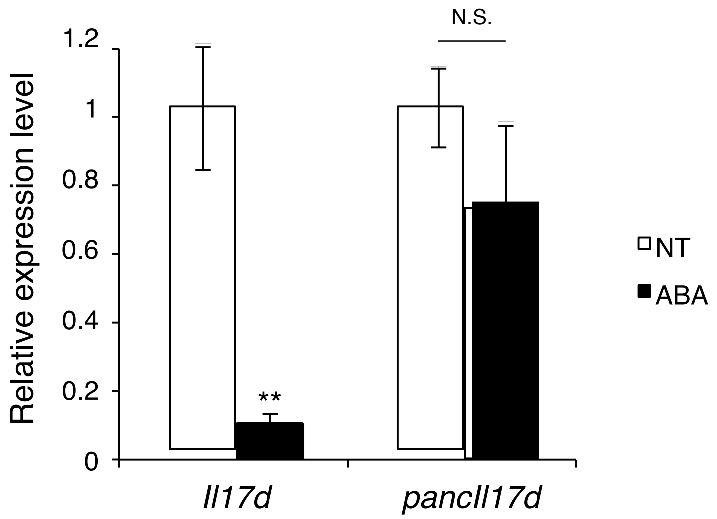

Hamazaki_Fig. S15

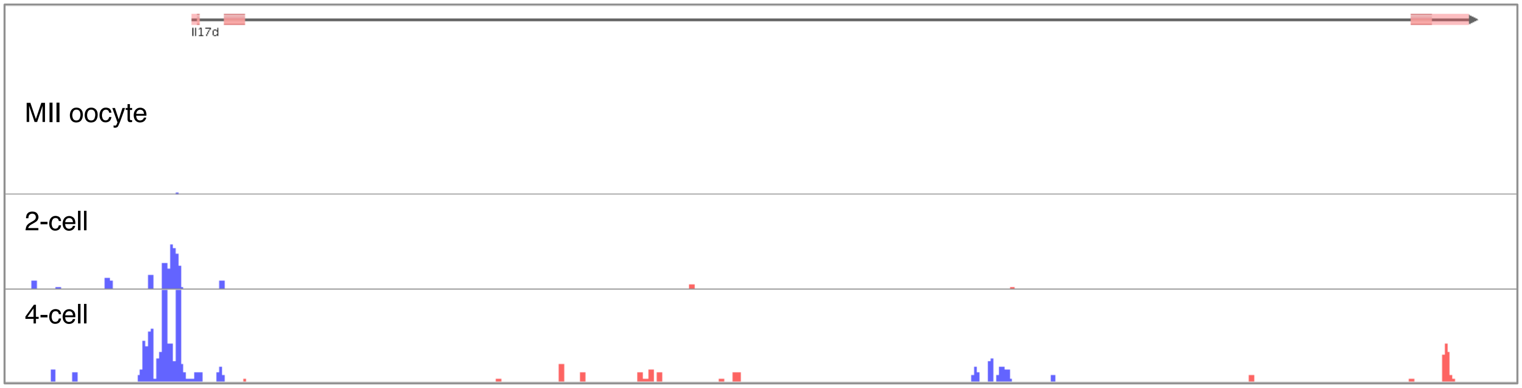

Hamazaki_Fig. S16

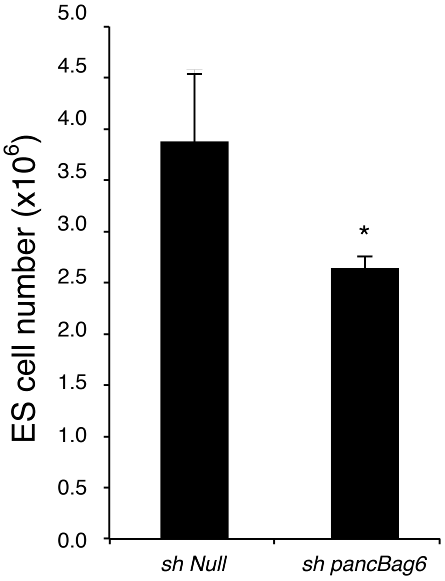

Table S1

Click here to Download Table S1

Table S2

Click here to Download Table S2

Table S3

Click here to Download Table S3

Table S4

Click here to Download Table S4

Table S5

Click here to Download Table S5

Table S6 List of the primers used in this study

**Bisulfite *Il17d* forward**	TTAAGTTGTAAGGTTTGAAGGGAT
**Bisulfite *Il17d* reverse**	ACTTACTCACTTACTTATTC
**Bisulfite *Il17d* TSS forward**	TAGTTGAATAAGAGGTATGGAG
**Bisulfite *Il17d* TSS reverse**	CTCACCAATATCCCCAACATCA
**Bisulfite *Mospd3* forward**	AGTGGGAAGAATGTAGTTTTTATTGTT
**Bisulfite *Mospd3* reverse**	TCTTACCCACAATTTCACTTAAAAAA
**Bisulfite *Tbc1d22a* forward**	ATGTATTTTATAATTAAGTATATTTTATTG
**Bisulfite *Tbc1d22a* reverse**	CTATAATATAATCCAAACCTCATC
**qPCR *pancMospd3* forward**	CTGCGGGAACTCAACATCAC
**qPCR *pancMospd3* reverse**	CTTGGGCAGAATCCACCTCT
**qPCR *Mospd3* forward**	TGATCTCCCCCTGTCCTCTTT
**qPCR *Mospd3* reverse**	AGTTCCCGTGGGGTTGTAGA
**qPCR *pancIl17d* forward**	GGAAAACAGCCTCCTTCTAGCC
**qPCR *pancIl17d* reverse**	TCTCCTTCTTGCGACCACTTC
**qPCR *Il17d* forward**	TCACACACATCCCGTTTTCC
**qPCR *Il17d* reverse**	CCGGAGCACTCATTATCACC
**qPCR *pancTbc1d22a* forward**	TCGTGTCTCCGTGCCTATTC
**qPCR *pancTbc1d22a* reverse**	TTCTCCCGCTGTAGTGTGGT
**qPCR *Tbc1d22a* forward**	TTGTGCTGCTTTCCTCGTGA
**qPCR *Tbc1d22a* reverse**	AGGCAGAGAGGGGATGCTAT
**qPCR *Oct3/4* forward**	GGCGTTCTCTTTGGAAAGGTGTTC
**qPCR *Oct3/4* reverse**	CTCGAACCACATCCTTCTCT
**qPCR *Sox2* forward**	CACAGATGCAACCGATGCA
**qPCR *Sox2* reverse**	GGTGCCCTGCTGCGAGTA
**qPCR *c-Myc* forward**	ACGACAGCAGCTCGCCCAAATC
**qPCR *c-Myc* reverse**	TGGAGCACTTGCGGTTGTTGCT
**qPCR *Klf4* forward**	ACCTGGCGAGTCTGACATGGCT
**qPCR *Klf4* reverse**	AGGATGAAGCTGACGCCGAGGT
**qPCR *Cdh1* forward**	CGACCGGAAGTGACTCGAAA
**qPCR *Cdh1* reverse**	AACCACTGCCCTCGTAATCG
**qPCR *Otx1* forward**	TGCCATGGACCTCCTGCACC
**qPCR *Otx1* reverse**	GTTCCATTCCCGCTCTGCTG
**qPCR *T* forward**	GCTTCAAGGAGCTAACTAACGAG
**qPCR *T* reverse**	CCAGCAAGAAAGAGTACATGGC
**qPCR *Gata6* forward**	TCATTACCTGTGCAATGCATGCGG
**qPCR *Gata6* reverse**	ACGCCATAAGGTAGTGGTTGTGGT

Table S7 List of siRNAs and shRNAs used in this study

si *pancIl17d_1*	GCUCAAAUGAAGGACUCUA
si *pancIl17d_2*	GCAUUUACGCUUUGAGAAU
si *pancMospd3*	UAAAUCUUUCCAGAAAAUCCA
si *pancTbc1d22a*	UUAACAUUUCCGUAUUAAGAU
si *Tet2*	GGAUGUAAGUUUGCCAGAAGC
si *Tet3*	GCUCCAACGAGAAGCUAUUUG
si *Il17d*	CCGAACACUACAUCACCAUTT
sh *pancMospd3* Fw	TGAAACTCTGGGAATTCAAAATTTCAAGAGAATTTTGAATTCCCAGAGTTTCTTTTTGGAAC
sh *pancMospd3* Rv	TCGAGTTCCAAAAAGAAACTCTGGGAATTCAAAATTCTCTTGAAATTTTGAATTCCCAGAGTTTCA
sh *pancTbc1d22a* Fw	TGGAAATGTTAATTATAAGTTTTTCAAGAGAAAACTTATAATTAACATTTCCTTTTTTGGAAC
sh *pancTbc1d22* Rv	TCGAGTTCCAAAAAAGGAAATGTTAATTATAAGTTTTCTCTTGAAAAACTTATAATTAACATTTCCA
sh *pancBag6* Fw	TGTAATTTCCTCGAGAAAAATTTTCAAGAGAAATTTTTCTCGAGGAAATTACTTTTTTGGAAC
sh *pancBag6* Rv	TCGAGTTCCAAAAAAGTAATTTCCTCGAGAAAAATTTCTCTTGAAAATTTTTCTCGAGGAAATTACA
